# In silico prediction of structure and function for a large family of transmembrane proteins that includes human Tmem41b

**DOI:** 10.12688/f1000research.27676.2

**Published:** 2021-03-25

**Authors:** Shahram Mesdaghi, David L. Murphy, Filomeno Sánchez Rodríguez, J. Javier Burgos-Mármol, Daniel J. Rigden

**Affiliations:** 1 Institute of Systems, Molecular and Integrative Biology, University of Liverpool, Liverpool, L69 7ZB, UK

**Keywords:** ab initio modelling, bioinformatics, autophagy, contact predictions, evolutionary covariance, DedA, SARS-CoV-2, Tmem41b, VTT domain

## Abstract

**Background:** Recent strides in computational structural biology have opened up an opportunity to understand previously uncharacterised proteins.  The under-representation of transmembrane proteins in the Protein Data Bank highlights the need to apply new and advanced bioinformatics methods to shed light on their structure and function.  This study focuses on a family of transmembrane proteins containing the Pfam domain PF09335 ('SNARE_ASSOC'/ ‘VTT ‘/’Tvp38’/'DedA'). One prominent member, Tmem41b, has been shown to be involved in early stages of autophagosome formation and is vital in mouse embryonic development as well as being identified as a viral host factor of SARS-CoV-2.

**Methods:** We used evolutionary covariance-derived information to construct and validate *ab initio *models, make domain boundary predictions and infer local structural features.

**Results:** The results from the structural bioinformatics analysis of Tmem41b and its homologues showed that they contain a tandem repeat that is clearly visible in evolutionary covariance data but much less so by sequence analysis.  Furthermore, cross-referencing of other prediction data with covariance analysis showed that the internal repeat features two-fold rotational symmetry. *Ab initio* modelling of Tmem41b and homologues reinforces these structural predictions.  Local structural features predicted to be present in Tmem41b were also present in Cl ^-^/H ^+ ^antiporters.

**Conclusions:** The results of this study strongly point to Tmem41b and its homologues being transporters for an as-yet uncharacterised substrate and possibly using H ^+^ antiporter activity as its mechanism for transport.

## Introduction

A protein’s structural information is crucial to understand it’s function and evolution. Currently, there is only experimental structural data for a tiny fraction of proteins ( Khafizov *et al.*, 2014). For instance, membrane proteins are encoded by 30% of the protein-coding genes of the human genome ( Almén *et al*., 2009), but they only have a 3.3% representation in the Protein Data Bank (PDB) (5785 membrane proteins out of 174507 PDB entries). Membrane protein families are particularly poorly understood due to experimental difficulties, such as over-expression, which can result in toxicity to host cells ( Grisshammer & Tateu, 1995), as well as difficulty in finding a suitable membrane mimetic to reconstitute the protein. Additionally, membrane proteins are much less conserved across species compared to water-soluble proteins ( Sojo *et al*., 2016), making sequence-based homologue identification a challenge, and in turn rendering homology modelling of these proteins more difficult. Membrane proteins can be grouped according to their interaction with various cell membranes: integral membrane proteins (IMPs) are permanently anchored whereas peripheral membrane proteins transiently adhere to cell membranes. IMPs that span the membrane are known as transmembrane proteins (TMEMs) as opposed to IMPs that adhere to one side of the membrane ( Fowler & Coveney, 2006). Membrane proteins also include various lipid-modified proteins ( Resh, 2016).

One IMP protein family is Tmem41, which has two human representatives, namely Tmem41a and Tmem41b; both share the PF09335 ('SNARE_ASSOC'/ ‘VTT ‘/’Tvp38’/‘DedA’) Pfam ( El-Gebali *et al*., 2019) domain. The profile of Tmem41b has recently risen due to experimental evidence pointing to its involvement in macroautophagy regulation (making it a possible Atg protein, i.e. an autophagy related protein) and lipid mobilisation ( Moretti *et al*., 2018). Other studies identify Tmem41b to be involved in motor circuit function, with TMEM41B-knockout *Drosophila* showing neuromuscular junction defects and aberrant motor neuron development in knockout zebrafish ( Lotti *et al*., 2012). Also, it has been reported that in TMEM41B-knockout HeLa cells there is an inhibition of Zika virus replication ( Scaturro *et al*., 2018). Tmem41b has also been identified as a host cell factor for SARS-CoV-2 ( Schneider *et al*., 2020). Tmem41b is the only common host cell factor identified for flaviviruses and coronaviruses and is the only autophagy-related protein identified as a viral host factor ( Hoffmann *et al*., 2021).

Additionally, Tmem41b has been shown to be essential for mouse embryonic development: homozygous knockout mice embryos suffer early termination of their development after 7–8 weeks ( Van Alstyne *et al*., 2018). Tmem41b is a structurally uncharacterised 291-residue protein found in the endoplasmic reticulum (ER) localising at the mitochondria-associated ER membranes ( Moretti *et al*., 2018). Disruption of the PF09335 domain by various residue substitutions ( Tábara *et al*., 2019) or its removal ( Morita *et al*., 2018) results in inhibition of autophagosome formation and impaired lipid mobilisation in human embryonic kidney (HEK) cells.

Tmem41b homologues, hereafter referred to as DedA proteins ( Morita *et al*., 2019), are present in all domains of life ( Keller & Schneider, 2013). The Pfam PF09335 domain was first identified in the *Saccharomyces cerevisiae* protein Tvp38 ( Inadome *et al*., 2007), and the authors concluded that Tvp38 associates with the tSNAREs in Tlg2-containing compartments, suggesting a role in membrane transport. Investigations into the bacterial and archaeal prevalence of these proteins showed that 90% of bacterial species and 70% of archaeal species encoded proteins with the PF09335 domain ( Doerrler *et al*., 2013). Bacterial and archaeal PF09335-containing proteins are collectively known as the DedA family ( Doerrler *et al.*, 2013; Nonet *et al.*, 1987). Detailed studies of the *Escherichia coli* DedA proteins have indicated that there are eight *E. coli* representatives of the DedA family (YqjA, YghB, YabI, YohD, DedA, YdjX, YdjZ, and YqaA) with overlapping functions ( Doerrler *et al*., 2013; Keller & Schneider, 2013), with YdjX and YdjZ being the most closely related to human Tmem41b in terms of sequence similarity ( Doerrler *et al*., 2013). Phenotypically, DedA knock-out *E. coli* cells display increased temperature sensitivity, cell division defects, activation envelope stress pathways, compromised proton motive force, sensitivity to alkaline pH and increased antibiotic susceptibility ( Doerrler *et al*., 2013; Keller *et al*., 2014). As *E. coli* expresses multiple DedA homologues, lethal effects are not observed as long as at least one DedA is expressed ( Kumar & Doerrler, 2014; Thompkins *et al*., 2008). *Borrelia burgdorferi* contains only one DedA protein in its genome and knockout cells display the same phenotype as the *E. coli* knockout strains. The *B. burgdorferi* homologue is indeed essential ( Liang *et al.*, 2010). Interestingly, *E. coli* knockout cells can be rescued with the *B. burgdorferi* homologue that shows only 19% sequence identity with YqjA. The functions of DedA have also been studied in the pathogen *Burkholderia thailandensis* where one family member was found to be required for resistance to polymyxin ( Panta *et al*., 2019).

Until the structure of poorly characterised protein families such as Pfam family PF09335 can be elucidated experimentally, *ab initio* protein modelling can be used to predict a fold allowing for structure-based function inferences ( Rigden *et al*., 2017). Such methods have made significant strides recently due to the availability of contact predictions ( Kinch *et al*., 2016). Prediction of residue-residue contacts relies on the fact that each pair of contacting residues covaries during evolution. The process of co-variation occurs as the properties of the two residues complement each other in order to maintain structural integrity of that local region and, consequently, its original functionality. Therefore, if one residue from the pair is replaced, the other must also change to compensate the physical chemical variation and hence preserve the original structure ( Lapedes *et al*., 1999). The link between two residues can be then reliably detected in multiple sequence alignments by using direct coupling analysis ( Morcos *et al*., 2011) as well as machine learning algorithms ( Wu *et al*., 2020). The predicted contacts can be used for a range of analyses such as the identification of domain boundaries ( Rigden, 2002; Simkovic *et al*., 2017a), but their main application is for contact-based modelling methods which can address larger targets than conventional fragment-assembly-based *ab initio* methods ( Yang *et al*., 2020). Contact-based modelling methods have been proven successful previously in modelling membrane proteins ( Hopf *et al*., 2012).

In the current study, we first linked the Pfam PF09335 family to the PF06695 family and chose a conveniently small Archaeal sequence and then utilised state of the art methods to make structural predictions for not only the Archaeal sequence but also for two prominent members of the Pfam family PF09335 (Tmem41b and YqjA) by exploiting data derived from sequence, evolutionary covariance and *ab initio* modelling. We are able to predict that both PF09335 homologues (DedA proteins) and PF06995 homologues contain re-entrant loops (stretches of protein that enter the bilayer but exit on the same side of the membrane) as well as a pseudo-inverted repeat topology. The predicted presence of both of these structural features strongly suggests that DedA proteins are secondary active transporters for an uncharacterised substrate.

## Methods

### Multiple Sequence Alignment

A multiple sequence alignment was generated using PSI/TM-COFFEE variant (RRID:SCR_019024) with default settings ( Floden *et al*., 2016).

### Pfam database screening

Searches using the sequences of DedA domain proteins Tmem41b, YqjA, YdjX, Ydjz, Tvp38 and Mt2055 were made against the Pfam-A_v32.0 (RRID:SCR_004726) ( El-Gebali *et al*., 2019) database using the HHPred (RRID:SCR_010276) v3.0 server ( Zimmermann *et al*., 2018) with default parameters (-p 20 -Z 10000 -loc -z 1 -b 1 -B 10000 -ssm 2 -sc 1 -seq 1 -dbstrlen 10000 -norealign -maxres 32000 -contxt /cluster/toolkit/production/bioprogs/tools/hh-suite-build-new/data/context_data.crf) and eight iterations for MSA generation in the HHblits ( Remmert *et al*., 2012) stage.

### Contact map predictions

The DeepMetapsicov v1.0 server ( Kandathil *et al*., 2019) was used to generate contact predictions with ConKit v0.12 ( Simkovic *et al*., 2017b) utilised to visualise the contact maps. ConPlot (RRID:SCR_019216) was used to overlay additional prediction data ( Sánchez Rodríguez *et al*., 2021).

### Other prediction data

Transmembrane helical topology predictions were obtained from the Topcons server ( Tsirigos *et al*., 2015). Secondary structure predictions were made employing a local installation of PSIPRED (RRID:SCR_010246) v4.0 ( McGuffin *et al*., 2000). ConKit was also used to predict and visualise potential structural domain boundaries ( Rigden, 2002; Simkovic *et al*., 2017a). Residue analysis of putative amphipathic regions were performed using HELIQUEST ( Gautier *et al*., 2008) to determine the presence, direction and magnitude of any hydrophobic moment. Residue conservation was determined using the Consurf server ( Ashkenazy *et al*., 2016).

### Dataset for custom re-entrant database

A library of re-entrant loop pdb structures together with the putative re-entrant loop structures from the query protein models were clustered on their structural similarity. The library was built by obtaining a non-redundant (removing redundancy with a 40% sequence identity threshold) set of 125 chains from the PDBTM (RRID:SCR_011962) ( Kozma *et al*., 2013) that contain at least one re-entrant loop. As this investigation focuses on re-entrant loops that are immediately preceded by a TM helix that is packed against the loop, all re-entrant loops (boundaries defined by PDBTM) in addition to the preceding 30 residues were extracted. The resulting 193 library entries ( https://figshare.com/articles/dataset/repository_zip/14055212), supplemented with the re-entrant loop features (defined by the OMP server ( Lomize *et al*., 2012) and accompanied by the preceding 30 residues) from the *ab initio* modelling underwent an all-against-all structural alignment using a local installation of Dali v4.0 ( Holm & Laakso, 2016). The Z-scores for these alignments were then used for clustering with CLANS v1.0 ( Frickey & Lupas, 2004) with a Z-score of 4.5 used as the cut-off threshold.

### Model building

*Ab initio* models were built using the trRosetta ( Yang *et al*., 2020) server with default settings. Conservation was mapped on to the models using the ConSurf server ( Ashkenazy *et al*., 2016). Visualisation of models was achieved using PyMOL (RRID:SCR_000305) v2.3.0 ( DeLano, 2002). 

### Structural alignments

Dali (RRID:SCR_013433) v4.0 ( Holm & Laakso, 2016) was used to structurally align the output models and to query against the PDBTM ( Kozma *et al*., 2013).

An earlier version of this article can be found on bioRxiv (doi: https://doi.org/10.1101/2020.06.27.174763)

## Results and discussion

### Sequence comparisons suggest Pfam families PF09335 and PF06695 are related

**Figure 1.**
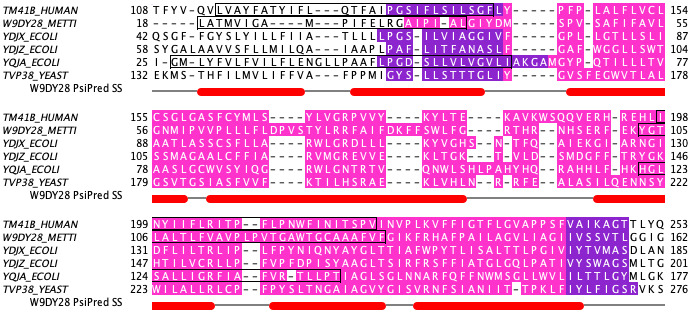
Multiple Sequence Alignment for query protein selection listed in Table 1. Magenta highlights the regions matched by HHpred to the PF09665 Pfam domain while purple is used for additional residues included in the PF09335 Pfam domain matches. The black boxed regions represent the locations of the putative re-entrant loops as identified by the modeling of the respective proteins. The secondary structure for the archaeal W9DY29 sequence (Mt2055) is also depicted with the relative positions of alpha helices shown as red blocks.

HHpred ( Zimmermann *et al*., 2018) was used to screen a selection of DedA proteins against the Pfam database ( El-Gebali *et al*., 2019). Hits were observed in the same region against both PF09335 and the Pfam domain PF06695 (‘Sm_multidrug_ex’) which is strongly indicative of homology: a probability of 99.4% with an E-value of 9E-17 for the PF09335 hit and 98.3% and 2E-10 respectively for PF06695. A HHpred search against the Pfam database using a member of PF06695 - the short archaeal sequence Mt2055 (UniProt code W9DY28) ( Apweiler *et al*., 2004) - returned similar results ( Table 1). Figure 1 shows the MSA for the same sequences along with the matched regions of the two Pfam domains under investigation. The Mt2055 sequence originates from the unpublished draft genome of the archaebacterium *Methanolobus tindarius* DSM 2278. For many of the subsequent analyses, the shorter archaeal sequence was used initially but the clear homology among this set of proteins means that inferences can be drawn across the group.

**Table 1.** HHpred results for Tmem41b and homologues demonstrate homology between Pfam families PF09335 and PF06695.

				PF09335 'SNARE_ASSOC'/ ‘VTT ‘/’Tvp38’ /DedA		PF06695 ‘Sm_multidrug_ex’	
	Species	UniProt Code	Length	Probability	E-Value	Probability	E-Value
Tmem41b	*Homo sapiens*	Q5BJD5	291	99.4	9E-17	98.3	2E-10
YdjX	*Escherichia coli*	P76219	236	99.6	2.1E-17	99.1	9.9E-13
Ydjz	*Escherichia coli*	P76221	235	99.6	1.1E-17	99.0	4.5E-16
YqjA	*Escherichia coli*	P0AA63	220	99.62	5.6E-15	99.41	1.3E-12
Tvp38	*Saccharomyces cerevisiae*	P36164	337	99.4	7.9E-15	98.7	2.7E-10
Mt2055	*Methanolobus tindarius*	W9DY28	168	99.0	2.4E-10	99.8	1.8E-20

There are no known experimental protein structures representing PF09335 or PF06695, but both Gremlin and DMPfold have constructed *ab initio* models for these Pfam domains ( Greener *et al*., 2019; Ovchinnikov *et al*., 2017).

### The predicted Pfam domains are inconsistent with a structural domain

**Figure 2.**
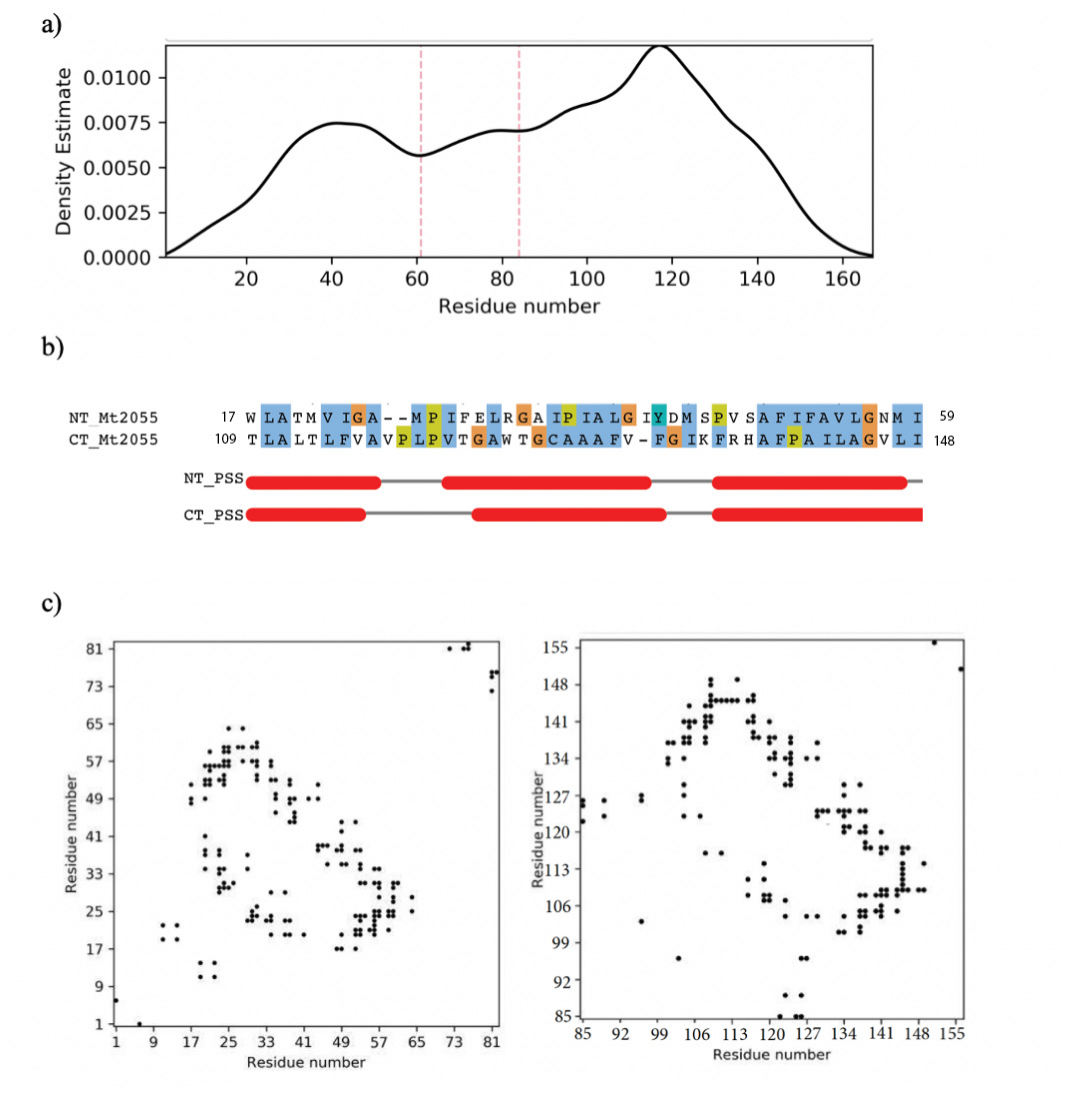
Mt2055 domain analysis. ( **a**) Contact density profile constructed by ConKit ( Simkovic *et al*., 2017b) utilising DeepMetaPSICOV contact prediction. Solid black line represents contact density and dotted red lines mark density minima corresponding to possible domain boundaries. ( **b**) HHalign alignments for the N-terminal and C-terminal Mt2055 halves, formatted using Jalview ( Waterhouse *et al*., 2009) and coloured according to the ClustalX scheme. Red bars represent helical secondary structure. ( **c**) Maps of predicted contacts generated by DeepMetaPSICOV and plotted using ConKit; left is N-terminal half (residues 1-84) and right is C-terminal half (residues 85-168). Black points represent predicted intramolecular contacts.

Analysis of the HHpred results obtained for the archaeal protein Mt2055 revealed the presence of additional hits for both PF06695 and PF09335 Pfam domains, in which the C-terminal half of the domains aligned with the N-terminal half of the Archaea protein. For example, residues 1-69 of the archaeal protein aligned with residues 52-117 of the Pfam PF09335 profile with a probability of 74.15%. Interestingly, contact density analysis ( Rigden, 2002; Sadowski, 2013) supported the existence of a domain boundary around residue 60, in broad agreement with the HHpred results ( Figure 2). Both the HHpred and contact density results therefore pointed to a specific domain structure being present.

### Sequence & contact prediction map analysis indicate that PF06695 is made up of a tandem repeat

When the Mt2055 sequence was split at residue 60-61, the resulting N-terminal region of 60 residues and the C-terminal section of 79 residues could be aligned using HHalign ( Soding, 2005) with a 78% probability and an E-value of 1.9E-3. Examination of the map of predicted contacts for Mt2055 reveals features that are present in both the N- and C-terminal halves of the protein ( Figure 2c). Taken together, these data strongly support the existence of a tandem repeat within the Mt2055 protein and hence across the PF06695 and PF09335 protein families.

**Figure 3.**
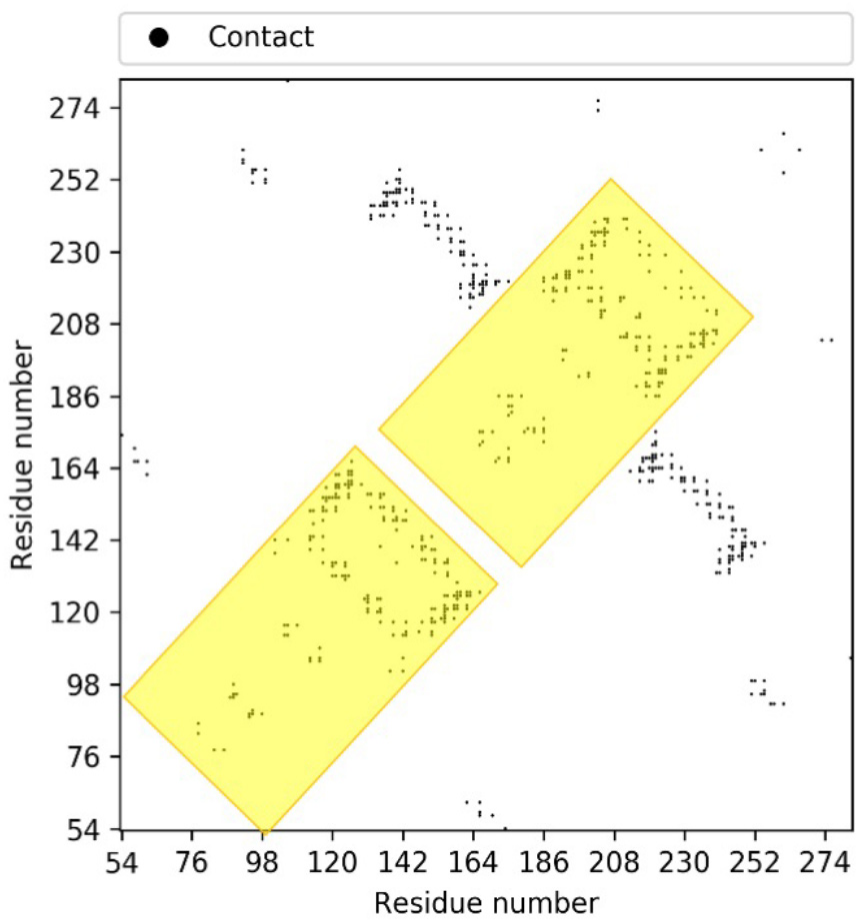
Tmem41b Contact map constructed using DeepMetaPSICOV and plotted using Conkit. The highlighted areas represent repeat units that have been revealed through evolutionary covariance analysis.

Interestingly, an equivalent sequence analysis with HHpred of other PF09335 homologues including Tmem41b itself does not reveal a repeat. However, inspection of their corresponding predicted contact maps does reveal features repeated when N- and C-halves of the protein are compared ( Figure 3). Apparently, evolutionary divergence has removed all trace of the repeat sequence signal in bacterial and eukaryotic proteins, although the feature remains visible by evolutionary covariance analysis.

### *Ab initio* modelling of Mt2055 reveals an unusual topology

Several authors have deposited structures of uncharacterised Pfam families in databases ( El-Gebali *et al*., 2019); however, Pfam domain boundaries for PF09335/PF06695, which define the limits of these previous modelling exercises, do not reflect the conserved structural domain that we predict. Given the fact that the available *ab initio* models were inconsistent with the transmembrane helix, secondary structure and contact predictions, we constructed our own models of Mt2055 as well as Tmem41b and YqjA with trRosetta. https://figshare.com/articles/dataset/repository_zip/14055212

**Figure 4.**
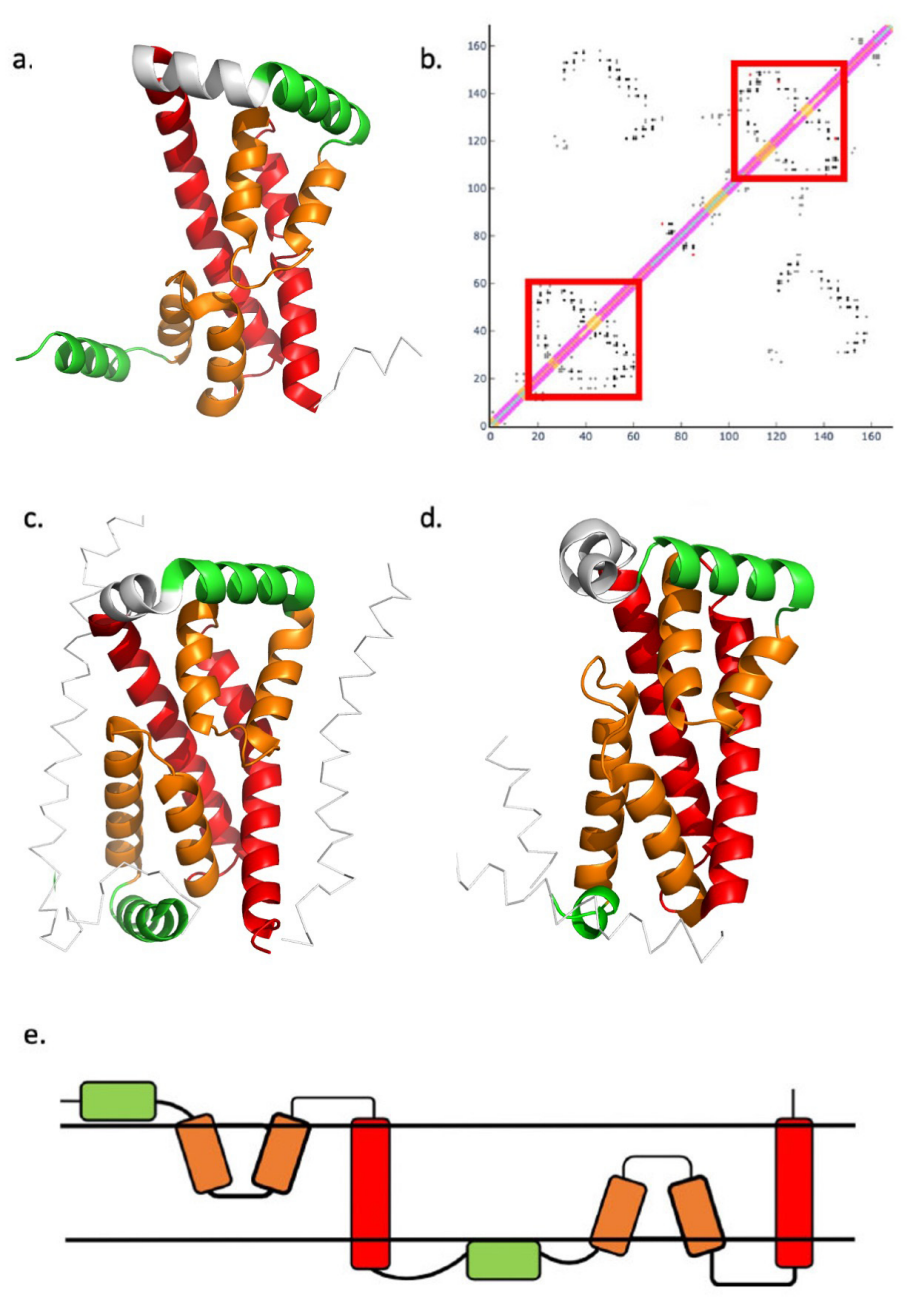
( **a**) trRosetta model of MT2055 - amphipathic helix (green) and a re-entrant loop (orange) packed with a TM helix (red) ( **b**) Superposition of DMP predicted contact map for Mt2055 and contacts from the Mt2055 model. Black points are matching contacts, red are mismatches and grey are contacts predicted but not present in the model. Diagonal is a visual representation of transmembrane helix and secondary structure prediction – central diagonal is the visualisation of the TopCons transmembrane prediction (orange being a TM helix) and the outer diagonals are the visual representation of the PSIPRED secondary structure prediction (pink – alpha helix and yellow – coil). Red boxes highlight the re-entrant loop and TM helix packing contact map signature. **c**) trRosetta model of Tmem41b only showing the conserved structural domain (residues 39-217) **d**) trRosetta model of YqjA only showing the conserved structural domain (residues 14-176). **e**) Proposed topology for (extended) DedA domain.

The Mt2055, Tmem41b and YqjA models had estimated TM scores from the trRosetta server of 0.633, 0.624 and 0.635 respectively, suggesting that they were likely to have captured the native fold of the family. All-against-all pairwise structural superposition of the models with DALI gave a mean Z-score of 11.9 confirming their strong similarity. We also used satisfaction of predicted contacts to validate the models ( Figure 4) ( Simkovic *et al*., 2017a). This showed that 80% of the top *L* predicted contacts (where *L* is the length of the protein) are satisfied by the model contacts for both Mt2055 and YqjA and a value of 60% was achieved for Tmem41b suggestive of good quality models ( de Oliveira *et al*., 2017).

The models ( Figure 3) contained interesting features: two inversely symmetrical repeated units each possessing a helix lying parallel to the membrane surface (green) and a re-entrant loop (orange) packed with a TM helix (red).

The presence of a re-entrant loop packed against each TM helix can also be seen on predicted contact maps for these proteins ( Figure 4b). Interestingly, each of the re-entrant helices are predicted as a single transmembrane region in the TopCons predictions. When cross-referenced with the PSIPRED secondary structure prediction it is noted that there is a predicted two-residue region of coil around the mid-point of the first TM helix prediction. A similar observation can be made for the fourth TM helix prediction with the equivalent coil region being six residues in length (see the diagonal of Figure 4b) Such a prediction would more obviously be treated as indicative of some kind of kink in the helix ( Law *et al.*, 2016) but the explanation here is that these regions form re-entrant helices. Similar contact map features, indicative of re-entrant loops packing against TM helices, can be seen clearly on the contact maps of other DedA proteins (data not shown). The MSA in Figure 1 shows the relative positions of the re-entrant loops in their respective sequences.

**Figure 5.**
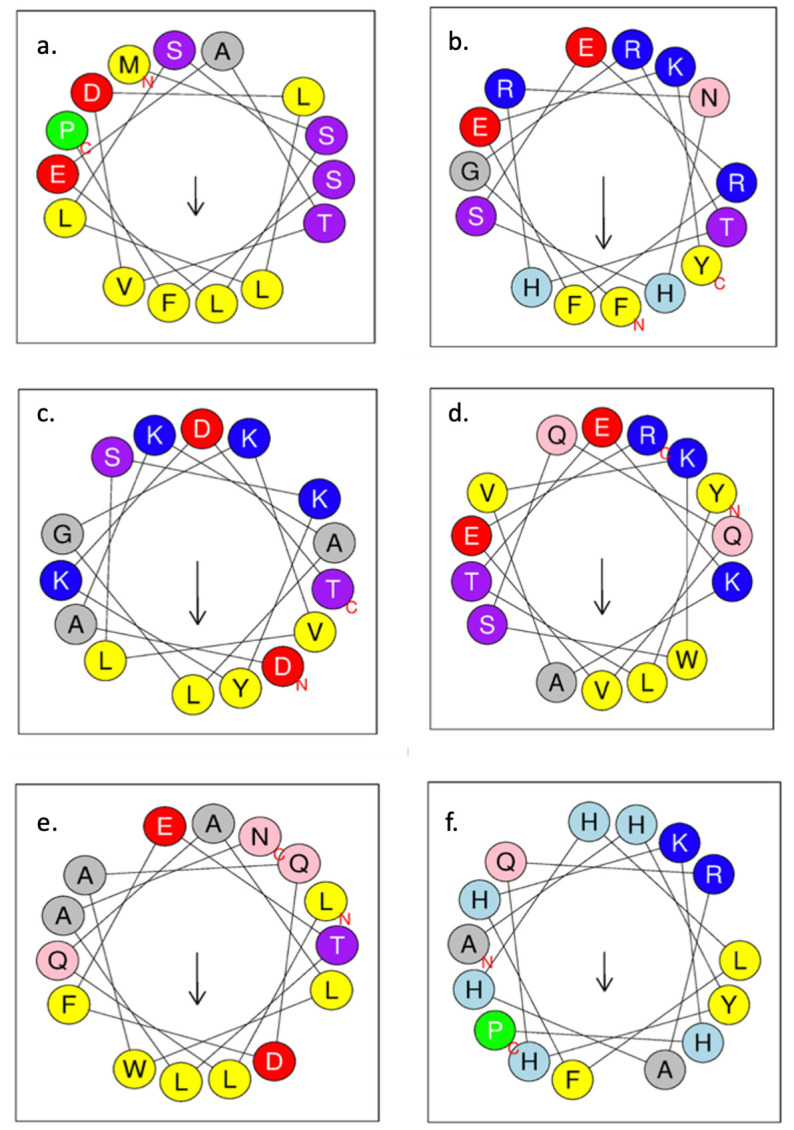
Helical wheel diagrams generated using the HELIQUEST server. Hydrophobic residues are shown in yellow, serine and threonine in purple, basic residues in dark blue, acidic residues in red, asparagine and glutamine in pink, alanine and glycine in grey, histidine in light blue and proline in green circles. Arrows represent direction and magnitude of the hydrophobic moment and residue marked with ‘N’ is the N-terminal end of the putative amphipathic helix with the residue marked ‘C’ being the C-terminal end. ( **a**) Mt2055 putative amphipathic helix 1 (hydrophobic moment of 0.298). ( **b**) Mt2055 putative amphipathic helix 2 (hydrophobic moment of 0.546). ( **c**) Tmem41b putative amphipathic helix 1 (hydrophobic moment of 0.471). ( **d**) Tmem41b putative amphipathic helix 2 (hydrophobic moment of 0.420). ( **e**). YqjA putative amphipathic helix 1 (hydrophobic moment of 0.295). ( **f**) YqjA putative amphipathic helix 2 (hydrophobic moment of 0.396).

In order to test for test whether the membrane-parallel helices (green in Figure 3) were amphipathic, an analysis of helical wheel diagrams for the fifteen residues preceding the putative re-entrant loops was performed with HELIQUEST ( Gautier *et al.*, 2008). The quantitative measures of the hydrophobic moment for the regions being analysed ( Figure 5) support that they are indeed amphipathic helices. The hydrophobic moments ranged from 0.298 to 0.546.

**Figure 6.**
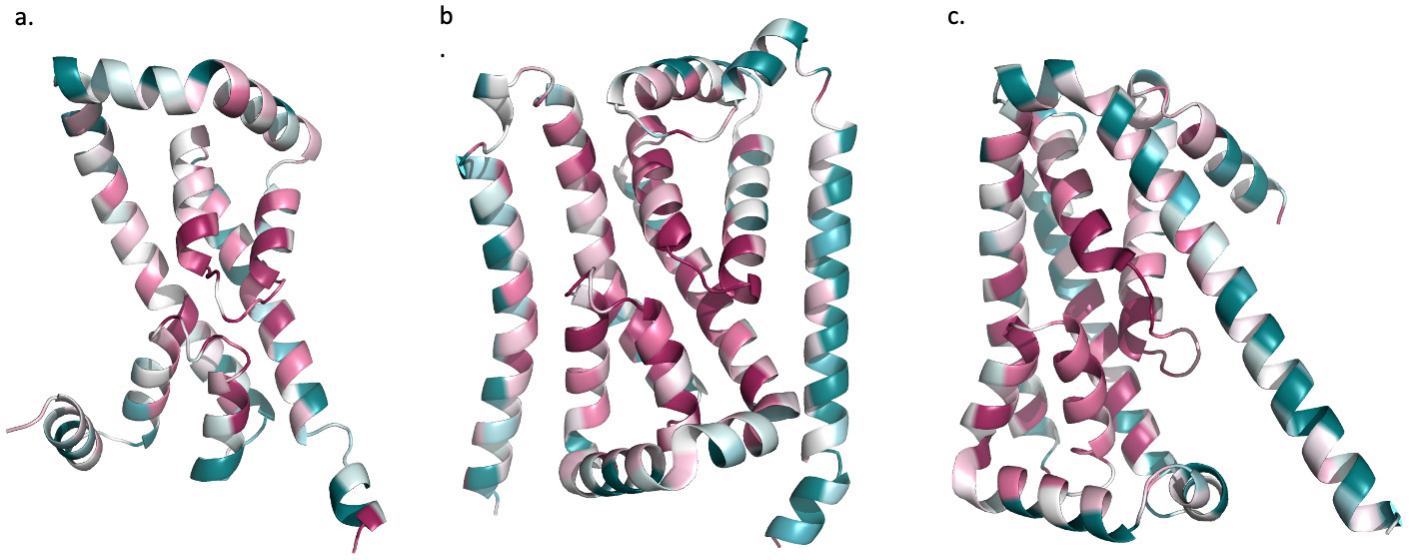
trRosetta models with Consurf conservation mapping for ( **a**) Mt2055 ( **b**) Tmem41b ( **c**) YqjA. Conservation is shown as a spectrum from purple (highly conserved) to blue (not conserved).

The predicted presence of the *amphipathic-re-entrant loop-TM helix* features in DedA domain proteins prompted a desire to map sequence conservation on to the ab initio models. Using the Consurf server to perform the mapping of sequence conservation onto the query models, it revealed that the re-entrant loop sequences are highly conserved. The high sequence conservation of re-entrant loops indicate that they are likely to be functionally and/or structurally important ( Figure 6).

### Re-entrant loops are also present in Cl ^-^/H ^+ ^Antiporters

The presence of re-entrant loops and the high density of conserved residues within them caused us to examine experimentally characterised re-entrant loops in the PDBTM database. A total of 193 non-redundant re-entrant helices were identified (see *Methods*). All 193 were clustered with the putative re-entrant loops from Mt2055, Tmem41b and YqjA using relative z-scores derived from an all-against-all DALI run and subsequently clustered in CLANS ( Frickey & Lupas, 2004) with a z-score cut-off of 4.5.

As expected all six re-entrant structures from the query models clustered together. The CLC transporter re-entrant structures of 3orgA (re-entrant 1 and re-entrant 2), 7bxu and 5tqq also clustered with the queries. Additionally, the re-entrant structure from an Undecaprenyl pyrophosphate phosphatase (UppP) (6cb2) also clustered with the queries. UppP is an integral membrane protein that recycles lipid and has structural similarities to CLC transporters ( Workman *et al.*, 2018). Contact maps derived from the pdb files of CLC and UppP structures show the contact map signature corresponding to the re-entrant/TM helix structural feature. Interestingly, the UppP is more similar to the query proteins being only 271 residues in length and having only 6 TM helices.

**Figure 7.**
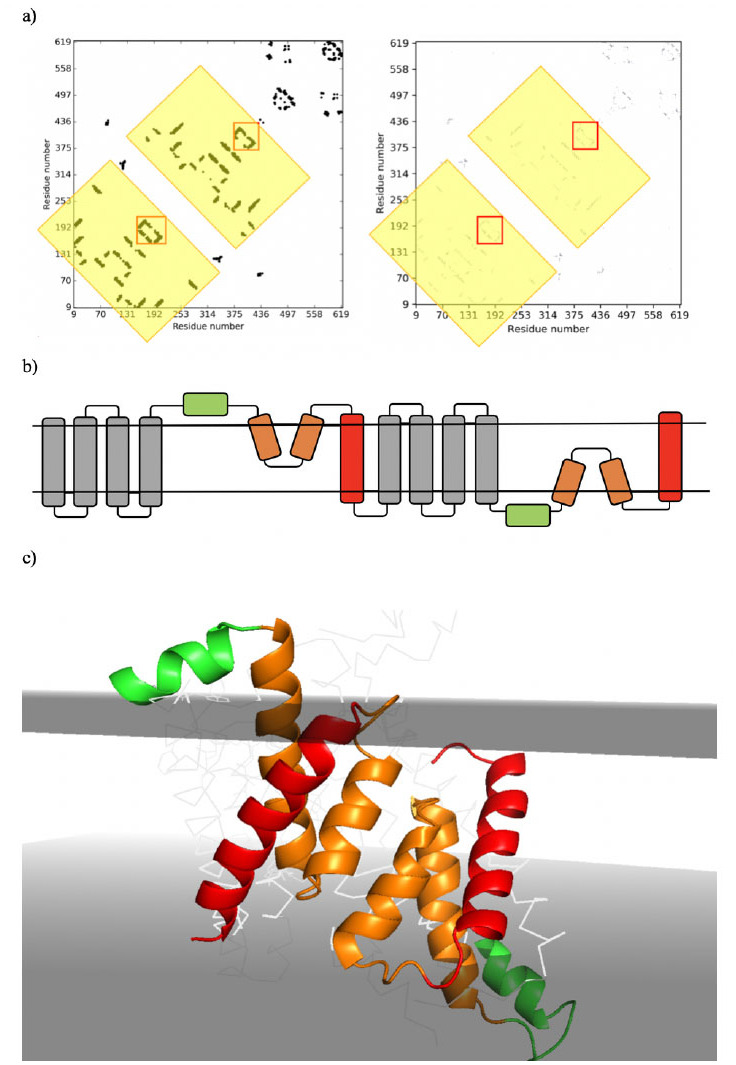
( **a**) Left - Predicted Contact map with repeating units highlighted in yellow boxes, contact map signature of re-entrant loop packed with TM helix in red boxes.; Right - The Experimental Contact map obtained from the PDB structure with repeating units highlighted in yellow boxes, contact map signature of re-entrant loop packed with TM helix in red boxes. ( **b**) Actual 3orgA topology; grey: TM Helices that are additional to the core; red: TM helices contributing to the formation of the core; orange; re-entrant loops contributing to the formation of the core; green: amphipathic helices contributing to the formation of the core. ( **c**) The 2-fold pseudo symmetry of the amphipathic/re-entrant loop/TM helix core inverted repeat structure of 3orgA with membrane positions shown as grey planes obtained from PDBTM.

Analysis of the Cl ^-^/H ^+ ^antiporter structures show that they contain a similar inverted repeat as we infer for the DedA homologues, resulting in pseudo-2-fold axis of symmetry running along the membrane ( Duran & Meiler, 2013). Again similarly, the Cl ^-^/H ^+ ^antiporter 3orgA also contains the amphipathic helices on the N-terminal side of the re-entrant loops. The fact that the presence of the amphipathic helices is restricted only to 3orgA and not found in all homologues suggest that these features are not essential for function ( Figure 7). 

### A possible antiporter role for DedA proteins

The presence of re-entrant loops in a transmembrane protein strongly indicates a transporter or pore functionality since this structural feature has, hitherto, only been found in proteins of this kind ( Yan & Luo, 2010). The structural similarities between the DedA proteins and the Cl ^-^/H ^+ ^antiporters raise the possibility that the families studied here are, in fact, unsuspected distant homologues having this putative pore feature in common. In that regard it is relevant to recall a hypothesis that DedA proteins are H ^+ ^antiporters resulting from site directed mutagenesis (SDM) experiments ( Kumar & Doerrler, 2014; Kumar *et al*., 2016). 

**Figure 8.**
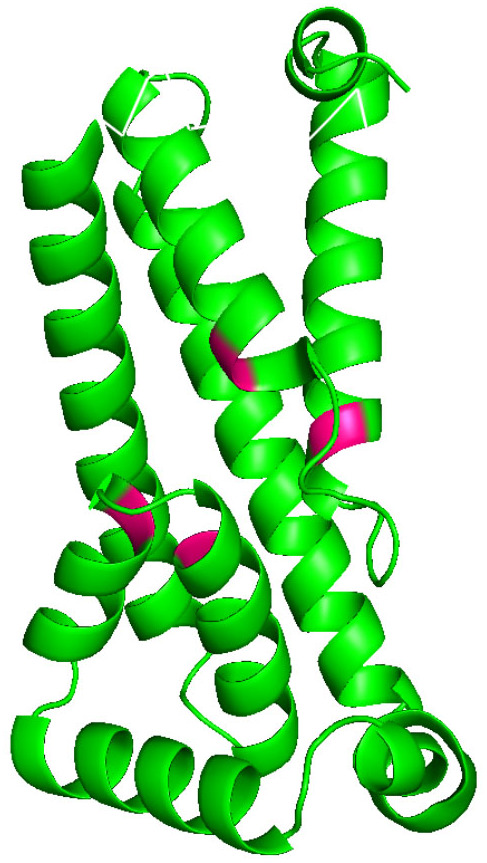
Essential residues determined by SDM experiments highlighted in pink on a truncated YqjA model.

A recent study has identified key residues ( Figure 8) in the *E. coli* DedA protein YqjA that, when replaced in site directed mutagenesis experiments, resulted in properly folded (membrane localized) but non-functional proteins unable to complement alkaline pH sensitivity of *E. coli* YqjA mutant and antibiotic sensitivity of YqjA/YghB double mutant ( Panta *et al*., 2019). Highlighting the essential residues (E39, D51, R130 and R136) on the YqjA model show that they come together in three-dimensional space with the N-terminal side of the first re-entrant possessing E39 and the C-terminal side possessing D51. R130 and R136 are similarly positioned on the second re-entrant loop ( Figure 8). Re-entrant loops are known to form pores and here we have two proton-titratable residues (E39, D51) in close proximity to essential basic residues (R130 and R136) within a putative pore. This three-dimensional arrangement of key residues could serve a role in the coupling of the protonation status with the binding of a yet to be characterised substrate as is postulated for the multi-drug H ^+ ^antiporter MdfA ( Heng *et al*., 2015) where these same residues are located inside a central cavity.

## Conclusions

This study demonstrates how covariance prediction data have multiple roles in modern structural bioinformatics: not just by acting as restraints for model making and serving for validation of the final models but by predicting domain boundaries and revealing the presence of cryptic internal repeats not evidenced by sequence analysis. Furthermore, we characterised a contact map feature characteristic of a re-entrant helix which may in future allow detection of this feature in other protein families.

Sequence, co-variance and *ab initio* modelling analyses show that the Pfam PF09335 and PF06695 domains are distantly homologous. These domains contain a structural core composed of a pseudo-inverse repeat of an amphipathic helix, a re-entrant loop and a TM helix. All PF09335 homologues contain this central core with additional TM- helices flanking either side. 

Querying the models against the PDB using Dali did not yield any significant hits. However, analysis of the prediction data revealed two features of DedA proteins that independently suggest that they are secondary transporters: both an inverted repeat architecture and the presence of a re-entrant loop, which are both independently and strongly associated with transporter function ( Duran & Meiler, 2013; Yan & Luo, 2010). Additionally, the fact that DedA proteins show structural similarities with H ^+^ antiporters indicate that these proteins may also couple substrate transport with an opposing H ^+^ current. Indeed, the YqjA homologue also contains strategically placed residues known to be involved in H ^+^ antiporter activity. The *ab initio* models show that the essential residues come together in the region that would be buried in the membrane potentially forming a substrate chamber consistent with the transport of a specific substrate. Further research needs to be carried out to determine what this substrate is and confirm the mechanism of transport.

## Data availability

Figshare: Final models and a list of PDB structures used for the clustering exercise https://doi.org/10.6084/m9.figshare.14055212.v1 ( Mesdaghi, 2021)
