# Supplementary material for: In silico prediction of structure and function for a large family of transmembrane proteins that includes human Tmem41b
Source: F1000Res. 2021 Mar 25;9:1395. Originally published 2020 Dec 3. [Version 2] doi: 10.12688/f1000research.27676.2 (PMC7818093; doi:10.12688/f1000research.27676.2)
Supplement: Peer review history

# Peer review: Reviewer response for version 2

**DOI:** 10.5256/f1000research.55392.r82225

**Published:** 2021-04-09

**Authors:** Claudio Bassot

**Affiliations:** 1 Department of Biochemistry and Biophysics, Science for Life Laboratory, Stockholm University, Stockholm, Sweden

Thank you to the authors for address the points I raised in my review. However, there is still a small typo in the first correction.

*The figure mentioned should be Figure 4, not Figure 3.*

*“The models ( Figure 3) contained interesting features: two inversely symmetrical repeated units each possessing an amphipathic helix lying parallel to the membrane surface (green) and a re-entrant loop (orange) packed with a TM helix (red).”*

*…..*

 “ *In order to test whether the membrane-parallel helices (green in Figure 3) were amphipathic, an analysis of helical wheel diagrams for the fifteen residues preceding the putative re-entrant loops was performed by with HELIQUEST ( Gautier et al., 2008).  The quantitative measures of the hydrophobic moment for the regions being analysed ( Figure 5) support that they are indeed amphipathic helices. The hydrophobic moments ranged from 0.298 to 0.546.”*

Is the work clearly and accurately presented and does it cite the current literature?

Partly

If applicable, is the statistical analysis and its interpretation appropriate?

Yes

Are all the source data underlying the results available to ensure full reproducibility?

No

Is the study design appropriate and is the work technically sound?

Yes

Are the conclusions drawn adequately supported by the results?

Yes

Are sufficient details of methods and analysis provided to allow replication by others?

Yes

Reviewer Expertise:

Modelling of transmembrane proteins.

I confirm that I have read this submission and believe that I have an appropriate level of expertise to confirm that it is of an acceptable scientific standard.

# Peer review: Reviewer response for version 2

**DOI:** 10.5256/f1000research.55392.r82226

**Published:** 2021-04-07

**Authors:** Gábor Tusnády, László Dobson

**Affiliations:** 1 Institute of Enzymology, Research Centre for Natural Sciences, Budapest, Hungary

All but one of our previous comments were responded. We accepted all responses, but authors should responded to this point too:

 "The most serious one: As it can be seen on Fig7b-c, 3org contains additional helices that surround the interfacial helix - re-entrant loop - tm structure. Indeed the protein are dimer, where the dimer interface are formed by the re-entrant loops and the additional transmembrane helices surround this core. This arrangement ensure the lipid embedded structure is energetically stable. In the proposed model, re-entrant loops are not wrapped by other helices thus lipids may interact them. This is energetically unfavorable and does not prefer for the suggested function too. The validity of the model should be further investigate by molecular dynamic simulations of lipid embedded structures."

Is the work clearly and accurately presented and does it cite the current literature?

Partly

If applicable, is the statistical analysis and its interpretation appropriate?

Partly

Are all the source data underlying the results available to ensure full reproducibility?

No

Is the study design appropriate and is the work technically sound?

Partly

Are the conclusions drawn adequately supported by the results?

Partly

Are sufficient details of methods and analysis provided to allow replication by others?

No

Reviewer Expertise:

Topology and structure prediction of transmembrane proteins.

We confirm that we have read this submission and believe that we have an appropriate level of expertise to confirm that it is of an acceptable scientific standard, however we have significant reservations, as outlined above.

# Peer review: Reviewer response for version 1

**DOI:** 10.5256/f1000research.30592.r75805

**Published:** 2021-01-20

**Authors:** Claudio Bassot

**Affiliations:** 1 Department of Biochemistry and Biophysics, Science for Life Laboratory, Stockholm University, Stockholm, Sweden

The authors model ab-initio Tmem41b and homologues, characterizing them as secondary transporters. The models are reliable and the study is scientifically robust and worthy of indexing. 

 However, two points are unclear in the text and need to be clarified, plus, some minor changes will improve the readability of the manuscript. Finally, the models could be made available to ensure full reproducibility.

 Major:

- The sentence: “The analysis was performed by HELIQUEST ( Gautier *et al.*, 2008) which constructed helical wheel diagrams and provided a quantitative measure of the hydrophobic moment for the region being analysed (Figure 4).” is out of context. In that paragraph are described the reentrant helices, shouldn’t the sentence (and the figure) be in the paragraph before where are mentioned the amphipathic helices? The figure discussion in the text should be extended.
- The role of Figure 7 is not clear. It is mentioned in the context of the description of the putative active residues but these are not shown in the figure. Moreover, the only reference to structure 3orgA (shown in the figure) is in the previous paragraph but it’s not related to Figure7. The authors should describe Figure 7 better.

Minor 

 Introduction:

- “but they only have a 2% representation in the Protein Data Bank (PDB) ( Koehler Leman *et al*., 2015)". The number of transmembrane proteins has grown significantly in the past few years. From the statistics of PDB and PDBTM the ratio of membrane proteins appears close to 4% now.

Results:

- Figure 1b could be clearer with the residues numbering on the sequences.
- Figure 5. The colours are misleading because they are the opposite of the standard consurf colouration (blue not conserved, purple conserved). The standard colouration would allow a faster understanding of the figure.

Is the work clearly and accurately presented and does it cite the current literature?

Partly

If applicable, is the statistical analysis and its interpretation appropriate?

Yes

Are all the source data underlying the results available to ensure full reproducibility?

No

Is the study design appropriate and is the work technically sound?

Yes

Are the conclusions drawn adequately supported by the results?

Yes

Are sufficient details of methods and analysis provided to allow replication by others?

Yes

Reviewer Expertise:

Modelling of transmembrane proteins.

I confirm that I have read this submission and believe that I have an appropriate level of expertise to confirm that it is of an acceptable scientific standard, however I have significant reservations, as outlined above.

# Response

**Published:** 2021-03-10

**Authors:** Daniel Rigden

**Affiliations:** University of Liverpool, UK

**The authors model ab-initio Tmem41b and homologues, characterizing them as secondary transporters. The models are reliable and the study is scientifically robust and worthy of indexing.**

**However, two points are unclear in the text and need to be clarified, plus, some minor changes will improve the readability of the manuscript. Finally, the models could be made available to ensure full reproducibility.**

 Models are now available as now mentioned in the text;

https://figshare.com/articles/dataset/repository_zip/14055212

**Major: **

- **The sentence: “The analysis was performed by HELIQUEST (** **Gautier *et al.*, 2008** **) which constructed helical wheel diagrams and provided a quantitative measure of the hydrophobic moment for the region being analysed (Figure 4).” is out of context. In that paragraph are described the reentrant helices, shouldn’t the sentence (and the figure) be in the paragraph before where are mentioned the amphipathic helices? The figure discussion in the text should be extended.**

Yes, we are in agreement with you, the paragraph did seem out of place as well as unfinished. In response we have re-worked the paragraph and noted the helices as merely membrane-parallel at first mention, deferring the question of amphipathicity.

*“The models ( Figure 3) contained interesting features: two inversely symmetrical repeated units each possessing an amphipathic helix lying parallel to the membrane surface (green) and a re-entrant loop (orange) packed with a TM helix (red).”*

*…..*

 “ *In order to test whether the membrane-parallel helices (green in Figure 3) were amphipathic, an analysis of helical wheel diagrams for the fifteen residues preceding the putative re-entrant loops was performed by with HELIQUEST ( Gautier et al., 2008).  The quantitative measures of the hydrophobic moment for the regions being analysed ( Figure 5) support that they are indeed amphipathic helices. The hydrophobic moments ranged from 0.298 to 0.546.”*

- **The role of Figure 7 is not clear. It is mentioned in the context of the description of the putative active residues but these are not shown in the figure. Moreover, the only reference to structure 3orgA (shown in the figure) is in the previous paragraph but it’s not related to Figure7. The authors should describe Figure 7 better.**

Thank you for pointing out the ambiguity of Figure 7.  There was an error in the figure numbering resulting in Figure 7 not being cited in the main text.  This has now been amended and the relevance of figure 7 is highlighted in the following paragraph;

 “ *Analysis of the Cl ^-^/H ^+ ^antiporter structures show that they contain a similar inverted repeat as we infer for the DedA homologues, resulting in pseudo-2-fold axis of symmetry running along the membrane (* *Duran & Meiler, 2013* *). Again similarly, the Cl ^-^/H ^+ ^antiporter 3orgA also contains the amphipathic helices on the N-terminal side of the re-entrant loops. The fact that the presence of the amphipathic helices is restricted only to 3orgA and not found in all homologues suggest that these features are not essential for function (Figure 7). “*

**Minor**

**Introduction: **

- **“but they only have a 2% representation in the Protein Data Bank (PDB) (** **Koehler Leman *et al*., 2015** **)". The number of transmembrane proteins has grown significantly in the past few years. From the statistics of PDB and PDBTM the ratio of membrane proteins appears close to 4% now.**

As suggested using the PDB & PDBTM stats we can see the ratio of membrane proteins is above the 2% previously quoted.  The PDBTM has 5785 entries with a total of 174507 entries for the PDB.  I have updated the introduction accordingly;

*“For instance, membrane proteins are encoded by 30% of the protein-coding genes of the human genome (* *Almén et al., 2009* *), but they only have a 3.3% representation in the Protein Data Bank (PDB) (5785 membrane proteins out of 174507 PDB entries).”*

**Results: **

- **Figure 1b could be clearer with the residues numbering on the sequences.**
  Yes agreed.  Numbering has been added to figure 1b (now 2b, due to the inclusion of an additional figure in the revised manuscript).  It can be seen that the additional detail of the numbering makes it easier to cross reference the images that make up this figure.  The new figure is shown;
- **Figure 5. The colours are misleading because they are the opposite of the standard consurf colouration (blue not conserved, purple conserved). The standard colouration would allow a faster understanding of the figure.**

We agree. Colouring on the B-factor column directly produces the results we show but the new blue-purple spectrum does seem to be well-adopted. We have therefore replaced the figure and updated the legend to read

*“trRosetta models with Consurf conservation mapping for (a) Mt2055 (b) Tmem41b (c) Yqja. Conservation is shown as a spectrum from purple (highly conserved) to blue (not conserved).”*

# Peer review: Reviewer response for version 1

**DOI:** 10.5256/f1000research.30592.r75806

**Published:** 2021-01-07

**Authors:** Gábor Tusnády, László Dobson

**Affiliations:** 1 Institute of Enzymology, Research Centre for Natural Sciences, Budapest, Hungary

In this manuscript Mesdaghi *et al*. describe the *in silico* structure modeling of three homologous integral membrane proteins Mt2055, Yqja and human Tmem41b. Structure determination of transmembrane proteins lacks behind globular ones for several reasons, giving space for computational tools. The proper use of these tools may unveil important structural aspects of transmembrane proteins but interpretation of results of such analysis should be done carefully. While the generated models in the manuscript are interesting and might be fully or partly true, the sequence analysis and interpretation of the results are problematic.

 Major:

 - The authors should be more specific about the exact boundaries of Pfam domains in different proteins as well as the sequence relations of proteins presented in Table 1. Please provide multiple sequence alignment for these proteins indicating the localization of the two pfam domains and the proposed re-entrant loops/transmembrane regions in the sequences.

 - The authors propose Mt2055 contains a tandem repeat and suggest the duplication is present in Tmem41b and Yqja structure as well even if it is undetectable from sequence analysis. The proposed domain boundary in Figure1a and arguments for tandem duplication does not seem convincing. The e-value of 1.9E-3 is quite large for the alignment. The authors should rule out that results in their paper may occur purely by chance. Please test the statistical significance of this value by generating pairwise alignments of transmembrane regions of unrelated transmembrane proteins with similar length. Moreover, contact maps for Mt2055 and Tmem41b were generated from the same multiple alignment, and therefore they must be identical/similar. Thus the similarities does not prove the tandem duplication occurred in Tmem41b too.

 - Structure modeling of membrane proteins is somewhat different from globular ones for several reasons. It is highly recommended to use specific software for this task or argue why used a non-specific one. On one hand, in general, topology prediction is more accurate than structure modeling and should be used as an input to aid the modeling. The reviewer is not sure the result of a standard *ab initio* structure modeling program is sufficient to question topology prediction results. On the other hand, topology prediction results are different for Tmem41b (6 TM helix) and Mt2055 (4 TM helix). Notably, other consensus topology method (CCTOP) have a similar result for Mt2055 (4 helix), but different for Tmem41b (6 helix). Using a third method (Octopus) a re-entrant loop is predicted. The authors should elaborate on such results instead of picking one method and running it on only one of the sequences.

 - Authors state: “For many of the subsequent analyses, the shorter archaeal sequence was used initially but the clear homology among this set of proteins means that inferences can be drawn across the group.” - Please provide the used multiple sequence alignment with pairwise similarities to support this statement.

 - It is not clear how helical wheels and hydrophobic moments support the manuscript - please provide a better description or omit these results.

 - Problems/Validation of re-entrant loops:

- The authors selected 56 sequence regions from PDBTM database and run an all-against-all Blast search and create clusters based on the search results. Since the sequence complexity of membrane regions are lowest than regions of globular proteins, the analysis should be repeated on randomly selected transmembrane segments. Please provide the list of the selected 56 re-entrant loops together with the results of the repeated analysis.
- Authors state: “The presence of a re-entrant loop packed against each TM helix can also be seen on predicted contact maps for these proteins (Figure 3b).” Re-entrant loops cannot be seen on contact map, only parallel and anti-parallel structures. A similar contact map can be easily generated from 3 transmembrane helices (1 parallel pair and two anti-parallel ones).
- The authors filtered removing any sequences of less than 10 residues and more than 20. Although the exact sequence localisation and length of the predicted re-entrant loop are not provided, the regions indicated as the “sign” of re-entrant loops on Figure 3b is larger than 20 residues and on the structures the orange regions contain 7 turns, thus the sequence length of them should be more than 20 residues (7*3.5=24.5).
- The most serious one: As it can be seen on Fig7b-c, 3org contains additional helices that surround the interfacial helix - re-entrant loop - tm structure. Indeed the protein are dimer, where the dimer interface are formed by the re-entrant loops and the additional transmembrane helices surround this core. This arrangement ensure the lipid embedded structure is energetically stable. In the proposed model, re-entrant loops are not wrapped by other helices thus lipids may interact them. This is energetically unfavorable and does not prefer for the suggested function too. The validity of the model should be further investigate by molecular dynamic simulations of lipid embedded structures.

- “The analysis was performed by HELIQUEST (Gautier *et al*., 2008) which constructed helical wheel diagrams and provided a quantitative measure of the hydrophobic moment for the region being analysed (Figure 4).” - This sentence and Figure 4 are pointless, containing data not used in the validation of the results.

 Minor:

- Abstract/Results: “The results from the structural bioinformatics analysis of Tmem41b and its homologues showed that they contain a tandem repeat that is clearly visible in evolutionary covariance data but much less so by sequence analysis.”
  - As I showed above, this statement might not be true. Moreover evolutionary covariance data is the results of sequence analysis, so this sentence is void of sense. Please rephrase.
- Introduction: “there are eight E. coli representatives of the DedA family (YqjA, YghB, YabI, Yoh, DedA, YdjX, YdjZ, and YqaA)”
  - Character D is missing in Yoh.
- Introduction: “In the current study, we utilised state of the art methods to make structural predictions for two prominent members of the Pfam family PF09335 (Tmem41b and Yqja) by exploiting data derived from sequence, evolutionary covariance and ab initio modelling.”
  - The most part of the manuscript deal with the sequence analysis of Mt2055, please rephrase this sentence in order to mirror this fact.
- "Interestingly, each of the re-entrant helices is predicted as a single transmembrane region in the TopCons predictions (see the diagonal of Figure 3b) with a two-residue region of coil in the centre."
  - TOPCONS does not predict coils and such details cannot be seen on the figure - please clarify this sentence.
- The authors should provide the generated PDB files as Supplementary Material.
- Contact map on Figure 7a left is the same that on right (numbering, dots). They should be different if one based on prediction and the other based on experimental data.

Is the work clearly and accurately presented and does it cite the current literature?

Partly

If applicable, is the statistical analysis and its interpretation appropriate?

Partly

Are all the source data underlying the results available to ensure full reproducibility?

No

Is the study design appropriate and is the work technically sound?

Partly

Are the conclusions drawn adequately supported by the results?

Partly

Are sufficient details of methods and analysis provided to allow replication by others?

No

Reviewer Expertise:

Topology and structure prediction of transmembrane proteins.

We confirm that we have read this submission and believe that we have an appropriate level of expertise to confirm that it is of an acceptable scientific standard, however we have significant reservations, as outlined above.

# Response

**Published:** 2021-03-10

**Authors:** Daniel Rigden

**Affiliations:** University of Liverpool, UK

**Major:**

**- The authors should be more specific about the exact boundaries of Pfam domains in different proteins as well as the sequence relations of proteins presented in Table 1. Please provide multiple sequence alignment for these proteins indicating the localization of the two pfam domains and the proposed re-entrant loops/transmembrane regions in the sequences.**

*Yes agreed, we agree that a MSA is useful. An MSA generated using PSI/TM-COFFEE has been added as Figure 1 to the manuscript.  The Pfam domains in questions well as the putative re-entrant loops for the modelled proteins have been highlighted to illustrate their relative positions.*

**- The authors propose Mt2055 contains a tandem repeat and suggest the duplication is present in Tmem41b and Yqja structure as well even if it is undetectable from sequence analysis. The proposed domain boundary in Figure1a and arguments for tandem duplication does not seem convincing. The e-value of 1.9E-3 is quite large for the alignment. The authors should rule out that results in their paper may occur purely by chance. Please test the statistical significance of this value by generating pairwise alignments of transmembrane regions of unrelated transmembrane proteins with similar length.**

*Utilisation of HHalign does result in an e-value of 1.9E-3 which on its own is not compelling. However, as highlighted, HHalign also expressed a probability score (a measure of statistical significance) of 78% which the software developers argue is a better indicator of significance than the e-value alone.*

*Additionally (as explained earlier in the text) ‘Analysis of the HHpred results (against Pfam database) obtained for the archaeal protein Mt2055 revealed the presence of additional secondary hits for both PF06695 and PF09335 Pfam domains, in which the C-terminal half of the domains aligned with the N-terminal half of the Archaea protein. For example, residues 1-69 of the archaeal protein aligned with residues 52-117 of the Pfam PF09335 profile with a probability of 74.15%.‘*

*It is important to remember that generating and scoring alignments with unrelated TM proteins is an intrinsic part of the database search. We have estimated that in Pfam there are currently around 1377 Pfam domains containing two or more TM-helices.  This is based on analysis of the Phobius predictions that are part of the* *current 33.1 r* *elease. In the search above, only three of these domains - PF08566, PF09835, PF13571 - scored comparably (* *probabilities of 73.3-76.6%) with the secondary hits* *for PF06695 and PF09335 . These results clearly place the secondary PF06695 and PF09335 matches at the extreme end of the score distribution for TM-helical Pfam domains, supporting their significance.*

*Arguably the above findings alone do not provide absolutely conclusive evidence of the presence of a repeat. However, reinforcing these findings we have the repeat that is revealed by the plotting of the predicted contacts and, consequently, the inverse repeat that is witnessed by the modelling.*

**Moreover, contact maps for Mt2055 and Tmem41b were generated from the same multiple alignment, and therefore they must be identical/similar. Thus the similarities does not prove the tandem duplication occurred in Tmem41b too.**

*Interesting point: however, we can confirm that the MSAs  used to generate the contacts maps for Mt2055 and Tmem41b were not identical.  The MSAs constructed by DMP were constructed independently using HHblits against the Uniprot database. The manuscript used the predicted contacts from the server and the MSAs generated to make the contact predictions are not made available to download with the results.  However, performing the contact prediction locally and utilising the same HHblits settings as the DMP server generates MSAs with 5000 sequences for each of the query proteins.  The predicted contact maps are very similar to those presented in the paper yet analysis reveals that the MSAs had only 1010 sequences in common.*

**- Structure modeling of membrane proteins is somewhat different from globular ones for several reasons. It is highly recommended to use specific software for this task or argue why used a non-specific one.**

*At the beginning of this project we had similar thoughts to you, therefore initially Rosetta membrane was utilised to build the models. However, the membrane protocol ‘forced’ TM helices where it was later clear from contact map analysis that re-entrant loops should be present.  Therefore, it was decided that contact restrained modelling software with proven success in regard to ab initio modelling of membrane proteins was used. Both DMPfold (local & server) as well as the trRosetta (server) models were constructed and similar folds were observed.  We note that the DMPfold paper benchmarked using transmembrane protein as explicitly says it ‘works just as well for transmembrane proteins.’ (https://www.nature.com/articles/s41467-019-11994-0 ). The trRosetta method was benchmarked against CASP13 targets which included transmembrane proteins (https://onlinelibrary.wiley.com/doi/abs/10.1002/prot.25775). The use of these covariance-based methods for membrane proteins has a long history so the following citation has been included in the revised manuscript;*

 Hopf, T. A., Colwell, L. J., Sheridan, R., Rost, B., Sander, C., & Marks, D. S. (2012). Three-dimensional structures of membrane proteins from genomic sequencing. *Cell*, *149*(7), 1607–1621.

**On one hand, in general, topology prediction is more accurate than structure modelling and should be used as an input to aid the modelling. The reviewer is not sure the result of a standard ab initio structure modelling program is sufficient to question topology prediction results. On the other hand, topology prediction results are different for Tmem41b (6 TM helix) and Mt2055 (4 TM helix). Notably, other consensus topology method (CCTOP) have a similar result for Mt2055 (4 helix), but different for Tmem41b (6 helix). Using a third method (Octopus) a re-entrant loop is predicted. The authors should elaborate on such results instead of picking one method and running it on only one of the sequences.**

*The different membrane topology prediction tools were used initially to predict the TMhelix boundaries for the query proteins.  We observed the same between the results of the different methods as yourself.  It was the variability of the topology predictions in addition to the contact map  features that led to the conclusion that something other than straightforward TM helices is present in the Pfam domains in question.  Indeed, TMHMM does show lower probability TMhelix predictions for the regions that the contact prediction and model making predict to be re-entrant loops.*

*To investigate further, visual representations of the membrane topology from TopCons and the psipred secondary structure prediction were plotted along the diagonal of the contact prediction for the query proteins.  This clearly highlights that the N and C halves of the predicted TM helices in question are making contact with each other (by a length of around 10 residues).  Additionally, the secondary structure plot shows an interruption at the halfway point of the predicted TM helices which would account for the abrupt change in direction of helix in the membrane.*

*Additionally, we have identified a crystal structure that is comparable in terms of size (293 residues) and has common structural features (inverted repeat with 2 re-entrant/TMhelix structures) to our query proteins; 6cb2.  For this protein, the TopCons topology prediction was compared to the actual topology of the crystal structure.*

*The above figure shows actual contacts for 6cb2 (black points) and a visual representation of the TopCons topology prediction (green -outside, red – TM helix, yellow-inside, yellow boxes are the re-entrant loop-TM-helix ‘signature’).  Cross-referencing the first re-entrant contact map  feature with the TopCons topology prediction it is clear that the TopCons topology must be wrong; the first TopCons predicted TM helix cannot be making contact with a region out-side of the membrane.  Indeed, examination of the crystal structure reveals that the contact feature highlighted does in fact result from a re-entrant loop packed with a TMhelix .*

*Furthermore, constructing ab initio models of 6cb2 using both trRosetta (server) and DMPfold (server) yielded models that correctly fold the re-entrant loop in question.  Performing structural alignments of the ab initio models against the crystal structure using Dali (server) give Z scores of 35 (trRosetta) and 27.5 (DMPfold).  These scores are leave no doubt that the correct fold has been modelled.  The image below shows the crystal structure in green and the trRosetta model in magenta.  The second image highlights the re-entrant feature that we are interested in.*

**- Authors state: “For many of the subsequent analyses, the shorter archaeal sequence was used initially but the clear homology among this set of proteins means that inferences can be drawn across the group.” - Please provide the used multiple sequence alignment with pairwise similarities to support this statement.**

*A multiple sequence alignment is now provided as Figure 1 and we note that all query sequences share the same Pfam domains so their homology is assured.*

**- It is not clear how helical wheels and hydrophobic moments support the manuscript - please provide a better description or omit these results.**

**- “The analysis was performed by HELIQUEST (Gautier et al., 2008) which constructed helical wheel diagrams and provided a quantitative measure of the hydrophobic moment for the region being analysed (Figure 4).” - This sentence and Figure 4 are pointless, containing data not used in the validation of the results.**

 Yes, we are in agreement with you, the paragraph did seem out of place as well as unfinished. In response we have re-worked the paragraph in question providing more clarity and analysis for the amphipathic analysis of the queries;

 “ *In order to test for the presence of the amphipathic helices, an analysis of helical wheel diagrams for the fifteen residues preceding the putative re-entrant loops was performed with HELIQUEST (* *Gautier et al., 2008* *).  The quantitative measures of the hydrophobic moment for the regions being analysed (Figure 5) support that they are indeed amphipathic helices. The hydrophobic moments ranged from 0.298 to 0.546.*”

 To clarify; from the helical wheel figures the amphipathic nature of the approximately 15 residues preceding the putative re-entrant loops is clear.  The importance of this finding is explained in relation to the structural comparison with the Cl ^-^/H ^+^ anti-porter 3org which also possesses the same structural features that we predict for the DedA proteins.

**-** **Problems/Validation of re-entrant loops:**

**•       The authors selected 56 sequence regions from PDBTM database and run an all-against-all Blast search and create clusters based on the search results. Since the sequence complexity of membrane regions are lowest than regions of globular proteins, the analysis should be repeated on randomly selected transmembrane segments. Please provide the list of the selected 56 re-entrant loops together with the results of the repeated analysis.**

•       The authors filtered removing any sequences of less than 10 residues and more than 20. Although the exact sequence localisation and length of the predicted re-entrant loop are not provided, the regions indicated as the “sign” of re-entrant loops on Figure 3b is larger than 20 residues and on the structures the orange regions contain 7 turns, thus the sequence length of them should be more than 20 residues (7*3.5=24.5).

**•       The most serious one: As it can be seen on Fig7b-c, 3org contains additional helices that surround the interfacial helix - re-entrant loop - tm structure. Indeed the protein are dimer, where the dimer interface are formed by the re-entrant loops and the additional transmembrane helices surround this core. This arrangement ensure the lipid embedded structure is energetically stable. In the proposed model, re-entrant loops are not wrapped by other helices thus lipids may interact them. This is energetically unfavorable and does not prefer for the suggested function too. The validity of the model should be further investigate by molecular dynamic simulations of lipid embedded structures.**

*Yes we are in agreement with you; the imposition of the re-entrant loop boundaries for the ab initio models were relatively arbitrary.  Therefore, the re-entrant loop screen against the PDBTM has been completely re-implemented.  Membrane boundaries for the models have now been predicted using the OMP server.  These boundaries provide the lengths of the putative re-entrant loops.  Consequently it is now recognised that the 20 residue ‘typical length’ of re-entrant loops may not be valid for the query models and the filtering of the larger loops for the clustering stage of this research had weak justification.*

*The clustering Methods text now reads*

*“A library of re-entrant loop pdb structures together with the putative re-entrant loop structures from the query protein models were clustered on their structural similarity.* *The library was built by obtaining a non-redundant (removing redundancy with a 40% sequence identity threshold) set of 125 chains from the PDBTM (RRID:SCR_011962) (* *Kozma et al., 2013* *)* *that contain at least one re-entrant loop.  As this investigation focuses on re-entrant loops that are immediately preceded by a TM helix that is packed against the loop, all re-entrant loops (boundaries defined by PDBTM) in addition to the preceding 30 residues were extracted.  The resulting 193 library entries (https://figshare.com/articles/dataset/repository_zip/14055212), supplemented with the re-entrant loop features (defined by the OMP server* *(Lomize, Pogozheva, Joo, Mosberg, & Lomize, 2012)* *and accompanied by the preceding 30 residues ) from the ab initio modelling underwent an all-against-all structural alignment using a local installation of Dali v4.0 (* *Holm & Laakso, 2016* *)* *. The Z-scores for these alignments were then used for clustering with* *CLANS* *v1.0 (* *Frickey & Lupas, 2004* *)* *with a Z-score of 4.5 used as the cut-off threshold.”*

*The Results of that protocol are now reported as follows*

*“The presence of re-entrant loops and the high density of conserved residues within them caused us to examine experimentally characterised re-entrant loops in the PDBTM database. A total of 193 non-redundant re-entrant helices were identified (see Methods). All 193 were clustered with the putative re-entrant loops from Mt2055, Tmem41b and YqjA using relative z-scores derived from an all-against-all DALI run and subsequently clustered in CLANS (* *Frickey & Lupas, 2004* *) with a z-score cut-off of 4.5.*

*The as expected all six re-entrant structures from the query models clustered together.  The CLC transporter re-entrant structures of 3orgA (re-entrant 1 and re-entrant 2), 7bxu and 5tqq also clustered with the queries. Additionally, the re-entrant structure from an Undecaprenyl pyrophosphate phosphatase (UppP) (6cb2) also clustered with the queries.  UppP is an integral membrane protein that recycles lipid and has structural similarities to CLC transporters* *(Workman, Worrall, & Strynadka, 2018)* *.  Contact maps derived from the pdb files of CLC and UppP structures show the contact map signature corresponding to the re-entrant/TM helix structural feature.  Interestingly, the UppP is more similar to the query proteins being only 271 residues in length and having only 6 TM helices.”*

*A list of the 125 chains from which the re-entrant structures were extracted from will be made available in a repository.*

**•       Authors state: “The presence of a re-entrant loop packed against each TM helix can also be seen on predicted contact maps for these proteins (Figure 3b).” Re-entrant loops cannot be seen on contact map, only parallel and anti-parallel structures. A similar contact map can be easily generated from 3 transmembrane helices (1 parallel pair and two anti-parallel ones).**

*Yes, a similar contact map feature can be easily generated from 3 transmembrane helices, however, this would result in a box feature of around 20x20 residues (and obviously reflected in the diagonal).  Since the re-entrant loop is making contact with itself this can only result in an approximately 10 residue antiparallel feature on the contact map.  Only approximately half of the TM helix that is packed with the re-entrant helix will be making contact with the re-entrant loop, therefore, this would result in an additional 10 residue antiparallel feature in addition to a 10-residue parallel feature.  Together with the diagonal these will display an approximately 10x10 box feature (also reflected in the diagonal) on the contact map rather than the 20x20 box feature that three transmembrane helices (1 parallel pair and two anti-parallel ones) would produce.  This can be seen below;*

**Minor:**

**•       Abstract/Results: “The results from the structural bioinformatics analysis of Tmem41b and its homologues showed that they contain a tandem repeat that is clearly visible in evolutionary covariance data but much less so by sequence analysis.”**

**- As I showed above, this statement might not be true. Moreover evolutionary covariance data is the results of sequence analysis, so this sentence is void of sense. Please rephrase.**

*We do not agree with this statement as sequence comparisons and co-variance comparisons are alternative methods to identify tandem repeats.  Yes, co-variance is derived from sequence analysis: however, co-variance data contains information that may not be present in data acquired from conventional sequence analysis.*

**•       Introduction: “there are eight E. coli representatives of the DedA family (YqjA, YghB, YabI, Yoh, DedA, YdjX, YdjZ, and YqaA)”**

**- Character D is missing in Yoh.**

*Thank you, corrected.*

**•       Introduction: “In the current study, we utilised state of the art methods to make structural predictions for two prominent members of the Pfam family PF09335 (Tmem41b and Yqja) by exploiting data derived from sequence, evolutionary covariance and ab initio modelling.”**

**- The most part of the manuscript deal with the sequence analysis of Mt2055, please rephrase this sentence in order to mirror this fact.**

*Thank you, the introduction has been updated to reflect the emphasis on the PF09665 Pfam domain and its representative Mt2055;*

 ‘ *In the current study, we first linked the Pfam PF09335 family to the PF06695 family and chose a conveniently small Archaeal sequence and then utilised state of the art methods to make structural predictions for not only the Archaeal sequence but also for two prominent members of the Pfam family PF09335 (Tmem41b and Yqja) by exploiting data derived from sequence, evolutionary covariance and ab initio modelling. We are able to predict that both PF09335 homologues (VTT proteins) and PF06995 homologues contain re-entrant loops (stretches of protein that enter the bilayer but exit on the same side of the membrane) as well as a pseudo-inverted repeat topology. The predicted presence of both of these structural features strongly suggests that VTT proteins are secondary active transporters for an uncharacterised substrate.’*

**•       "Interestingly, each of the re-entrant helices is predicted as a single transmembrane region in the TopCons predictions (see the diagonal of Figure 3b) with a two-residue region of coil in the centre."**

**- TOPCONS does not predict coils and such details cannot be seen on the figure - please clarify this sentence.**

*It was not our intention to suggest that Topcons predicts secondary structure.  We have changed the paragraph in question clarifying our intention;*

*"Interestingly, each of the re-entrant helices is predicted as a single transmembrane region in the TopCons predictions.  When cross-referenced with the PSIPRED secondary structure prediction it is noted that there is a predicted two-residue region of coil region of coil around the mid-point of the first TM helix prediction. A similar observation can be made for the fourth TM helix prediction with the equivalent coil region being six residues in length (see the diagonal of Figure 4b)"*

**•       The authors should provide the generated PDB files as Supplementary Material.**

 Thank you for pointing out this important omission. Since this journal does not allow Supplementary Material we have deposited the models in a repository now mentioned in the paper.

*https://figshare.com/articles/dataset/repository_zip/14055212*

**•       Contact map on Figure 7a left is the same that on right (numbering, dots). They should be different if one based on prediction and the other based on experimental data.**

*The left image was generated using predictions and the right with pdb file.  They are very similar, but this is to be expected.  ClC transporters are a large family and therefore the co-variance-derived predictions will be very accurate.*

# Peer review: Reviewer response for version 1

**DOI:** 10.5256/f1000research.30592.r75807

**Published:** 2020-12-17

**Authors:** William T. Doerrler, Pradip Panta

**Affiliations:** 1 Department of Biological Sciences, Louisiana State University, Baton Rouge, LA, USA

This work describes the computational structural modeling of a conserved membrane protein family that includes human TMEM41B, a protein with a number of reported functions. Membrane proteins are poorly represented in the structural database and computational methods are increasingly valuable for understanding structure and function. Here, they use a method using evolutionary amino acid contact co-variation to predict a structure that supports a proposed function as a proton dependent antiporter. While I am not fluent in the computational methods used, their prediction do align with published experimental work. The manuscript is well written and informative, but with a number of factual errors. I also suggest additional citations.

 I would like to begin with nomenclature. I received an email from Dr. Noburo Mizushima several months ago. He has published work on the TMEM41B protein. Also included on the email was Lucy Forrest, Dirk Schneider, and Rebecca Keller. It was Dr. Mizushima’s suggestion to name this protein family the "DedA superfamily" that includes both prokaryotic and eukaryotic proteins (DedA, VMP, and TMEM41 families). Accordingly, the shared domain will be called "DedA domain" and "VTT" domain would no longer be used. All recipients of this email agreed to using this nomenclature moving forward. Therefore, to avoid confusion, I would like the authors to adopt this nomenclature. I can forward the email upon request.

 Since the manuscript contains no line numbers, I will list the suggested corrections by paragraph:

**Introduction:**

 Paragraph 1: Formally, “membrane proteins” also include various lipid-modified proteins of both prokaryotes and eukaryotes in addition to integral and peripheral membrane proteins.

 Paragraph 4: “DedA” does not stand for “death effector domain”. It was named in a 1987 paper ^1^. See page 12213 of that article. I would like to see this article cited as well for historical purposes.

 The sentence that begins with “Phenotypically, DedA knockout E. coli…” should instead read “Phenotypically, E. coli lacking both *yqjA* and *yghB* (encoding proteins with 60% amino acid identity and partially overlapping functions)…." This paragraph should also cite ^2^. 

 The sentence that reads “As *E. coli *expresses multiple DedA homologues, the redundancy protects the cells from the phenotypical effects of single or multiple knock-outs as long as at least one DedA is expressed” should read “As *E. coli *expresses multiple DedA homologues, lethal effects are not observed as long as at least one DedA is expressed”. Cite the following article ^3^.

 You may also point out that the sole DedA family gene in *Borrelia burgdorferi* is indeed essential ^4^.

 YqjA is misspelled “YdjA”

 The sentence “Attempts to rescue….” Should be removed, as it does not make sense.

 The final sentence about Pseudomonas cites a non-peer reviewed proceeding abstract. I would like all citations to “Justice *et al.* 2016” removed from this article. This sentence can be replaced with the equally effective “The functions of DedA have also been studied in the pathogen *Burkholderia thailandensis* where one family member was found to be required for resistance to polymyxin” ^5^.

 Paragraph 6: “YqjA” is spelled “Yqja” here and throughout the manuscript and should be corrected. This includes in Table 1.

**Methods:**

 Paragraph 1: Please spell “Ydjx” and other bacterial proteins as “YdjX” with the final letter capitalized.

**Results and discussion:**

 Paragraph 5: first sentence, remove “however”.

 Paragraph 14: “A possible role for VTT proteins” final sentence remove “Justice *et al.*” and instead cite ^6^^,^^7^.

 Also, in this sentence, define “SDM” as “site directed mutagenesis”.

 Paragraph 15, first sentence. This statement is incorrect. Mutation of D51, E39, R130 or R136 in YqjA resulted in properly folded (membrane localized) but nonfunctional proteins unable to complement alkaline pH sensitivity of E. coli YqjA mutant and antibiotic sensitivity of YqjA/YghB double mutant.

 Finally, another interesting example of a membrane protein antiporter with re-entrant helices is the undecaprenyl pyrophosphate phosphatase UppP. It is up the authors if they would like to cite these articles ^8^^,^^9^.

Is the work clearly and accurately presented and does it cite the current literature?

Partly

If applicable, is the statistical analysis and its interpretation appropriate?

Yes

Are all the source data underlying the results available to ensure full reproducibility?

Yes

Is the study design appropriate and is the work technically sound?

Yes

Are the conclusions drawn adequately supported by the results?

Yes

Are sufficient details of methods and analysis provided to allow replication by others?

Yes

Reviewer Expertise:

Bacterial genetics with interests in membrane proteins and antibiotic resistance mechanisms.

We confirm that we have read this submission and believe that we have an appropriate level of expertise to confirm that it is of an acceptable scientific standard.

## References

[1] : The hisT-purF region of the Escherichia coli K-12 chromosome. Identification of additional genes of the hisT and purF operons. *J Biol Chem*.1987;262(25) :12209-17 3040734

[2] : Temperature sensitivity and cell division defects in an Escherichia coli strain with mutations in yghB and yqjA, encoding related and conserved inner membrane proteins. *J Bacteriol*.2008;190(13) : 10.1128/JB.00414-08 4489-500 10.1128/JB.00414-08 18456815PMC2446817

[3] : Multiple deletions reveal the essentiality of the DedA membrane protein family in Escherichia coli. *Microbiology (Reading)*.2012;158(Pt 5) : 10.1099/mic.0.056325-0 1162-1171 10.1099/mic.0.056325-0 22301910

[4] : BB0250 of Borrelia burgdorferi is a conserved and essential inner membrane protein required for cell division. *J Bacteriol*.2010;192(23) : 10.1128/JB.00571-10 6105-15 10.1128/JB.00571-10 20870761PMC2981195

[5] : A DedA Family Membrane Protein Is Required for Burkholderia thailandensis Colistin Resistance. *Front Microbiol*.2019;10: 10.3389/fmicb.2019.02532 2532 10.3389/fmicb.2019.02532 31827463PMC6849406

[6] : Members of the conserved DedA family are likely membrane transporters and are required for drug resistance in Escherichia coli. *Antimicrob Agents Chemother*.2014;58(2) : 10.1128/AAC.02238-13 923-30 10.1128/AAC.02238-13 24277026PMC3910893

[7] : Identification of essential arginine residues of Escherichia coli DedA/Tvp38 family membrane proteins YqjA and YghB. *FEMS Microbiol Lett*.363(13) : 10.1093/femsle/fnw133 10.1093/femsle/fnw133 27190159

[8] : Crystal structure of an intramembranal phosphatase central to bacterial cell-wall peptidoglycan biosynthesis and lipid recycling. *Nature Communications*.2018;9(1) : 10.1038/s41467-018-03547-8 10.1038/s41467-018-03547-8 PMC586105429559664

[9] : Crystal structure of undecaprenyl-pyrophosphate phosphatase and its role in peptidoglycan biosynthesis. *Nature Communications*.2018;9(1) : 10.1038/s41467-018-03477-5 10.1038/s41467-018-03477-5 PMC585202229540682

# Response

**Published:** 2021-03-10

**Authors:** Daniel Rigden

**Affiliations:** University of Liverpool, UK

**I would like to begin with nomenclature. I received an email from Dr. Noburo Mizushima several months ago. He has published work on the TMEM41B protein. Also included on the email was Lucy Forrest, Dirk Schneider, and Rebecca Keller. It was Dr. Mizushima’s suggestion to name this protein family the "DedA superfamily" that includes both prokaryotic and eukaryotic proteins (DedA, VMP, and TMEM41 families). Accordingly, the shared domain will be called "DedA domain" and "VTT" domain would no longer be used. All recipients of this email agreed to using this nomenclature moving forward. Therefore, to avoid confusion, I would like the authors to adopt this nomenclature. I can forward the email upon request.**

*Thanks very much for this helpful suggestion. We have changed the naming in the manuscript throughout to *“DedA superfamily”

**Introduction:**

**Paragraph 1: Formally, “membrane proteins” also include various lipid-modified proteins of both prokaryotes and eukaryotes in addition to integral and peripheral membrane proteins.**

*Thank you, this omission has been rectified;*

*‘Membrane proteins can be grouped according to their interaction with various cell membranes: integral membrane proteins (IMPs) are permanently anchored whereas peripheral membrane proteins transiently adhere to cell membranes. IMPs that span the membrane are known as transmembrane proteins (TMEMs) as opposed to IMPs that adhere to one side of the membrane (* *Fowler & Coveney, 2006* *).  Membrane proteins also include various lipid-modified proteins* *(Resh, 2016)* *.’*

**Paragraph 4: “DedA” does not stand for “death effector domain”. It was named in a 1987 paper** **^1^** **. See page 12213 of that article. I would like to see this article cited as well for historical purposes.**

*Your clarification on this nomenclature is appreciated.  We have amended the manuscript to reflect this error and included the reference for its historical importance.*

- **The sentence that begins with “Phenotypically, DedA knockout E. coli…” should instead read “Phenotypically, E. coli lacking both *yqjA* and *yghB* (encoding proteins with 60% amino acid identity and partially overlapping functions)…." This paragraph should also cite** **^2^** **. **
- **The sentence that reads “As *E. coli *expresses multiple DedA homologues, the redundancy protects the cells from the phenotypical effects of single or multiple knock-outs as long as at least one DedA is expressed” should read “As *E. coli *expresses multiple DedA homologues, lethal effects are not observed as long as at least one DedA is expressed”. Cite the following article** **^3^** **.**
- **Paragraph 14: “A possible role for VTT proteins” final sentence remove “Justice *et al.*” and instead cite** **^6^** **^,^** **^7^** **.**

*The additional references for previous experimental studies of the DedA proteins that you have suggested have been added to the manuscript.  Thanks for your suggestions which certainly make the manuscript more comprehensively cite previous studies.*

- **You may also point out that the sole DedA family gene in *Borrelia burgdorferi* is indeed essential** **^4^** **.**
- **The final sentence about Pseudomonas cites a non-peer reviewed proceeding abstract. I would like all citations to “Justice *et al.* 2016” removed from this article. This sentence can be replaced with the equally effective “The functions of DedA have also been studied in the pathogen *Burkholderia thailandensis* where one family member was found to be required for resistance to polymyxin”** **^5^** **.**

*Thanks for these helpful suggestions. The inclusion of these points brings more clarity to the paragraph in question;*

*“Borrelia burgdorferi* contains only one DedA protein in its genome and knockout cells display the same phenotype as the *E. coli* knockout strains.  The *B. burgdorferi* homologue is indeed essential (Liang et al., 2010). Interestingly, *E. coli* knockout cells can be rescued with the *B. burgdorferi* homologue that shows only 19% sequence identity with YqjA. The functions of DedA have also been studied in the pathogen *Burkholderia thailandensis* where one family member was found to be required for resistance to polymyxin (Panta et al., 2019).”

- **YqjA is misspelled “YdjA”**
- **Paragraph 6: “YqjA” is spelled “Yqja” here and throughout the manuscript and should be corrected. This includes in Table 1.**
- **P** **aragraph 1: Please spell “Ydjx” and other bacterial proteins as “YdjX” with the final letter capitalized.**

*Corrections made. Thank you for pointing out these important errors.*

**The sentence “Attempts to rescue….” Should be removed, as it does not make sense.**

 Sentence removed, thank you.

**Results and discussion:**

**Paragraph 5: first sentence, remove “however”.**

*Amendment made, thank you.*

**Also, in this sentence, define “SDM” as “site directed mutagenesis”.**

*Amendment made, thank you.*

**Paragraph 15, first sentence. This statement is incorrect. Mutation of D51, E39, R130 or R136 in YqjA resulted in properly folded (membrane localized) but nonfunctional proteins unable to complement alkaline pH sensitivity of E. coli YqjA mutant and antibiotic sensitivity of YqjA/YghB double mutant.**

*Thank you for pointing this out to us.  We can see our description of the results of the study was incorrect.  This has been amended with your direction;*

*“A recent study has identified key residues ( Figure* *8* *) in the E. coli DedA protein YqjA that, when replaced in site directed mutagenesis experiments, resulted in* *properly folded (membrane localized) but non-functional proteins unable to complement alkaline pH sensitivity of E. coli YqjA mutant and antibiotic sensitivity of YqjA/YghB double mutant* *(* *Panta et al., 2019* *).”*

**Finally, another interesting example of a membrane protein antiporter with re-entrant helices is the undecaprenyl pyrophosphate phosphatase UppP. It is up the authors if they would like to cite these articles** **^8^** **^,^** **^9^** **.**

*Yes, this is interesting; for the revision of the manuscript we re-implemented the re-entrant loop screen against the PDBTM.  We found that 6cb2 (UppP) re-entrant loop structures were structurally very similar to the re-entrant models for the DedA domains.*
